# Cutaneous Kaposi Sarcoma as a Presenting Sign of HIV: A Case Report

**DOI:** 10.7759/cureus.51043

**Published:** 2023-12-24

**Authors:** Simran Kalsi, Kaya L Curtis, Sarah Nocco, Linglei Ma, Shari Lipner

**Affiliations:** 1 Department of Medicine, Division of Dermatology, Larner College of Medicine, Burlington, USA; 2 Department of Dermatology, Weill Cornell Medicine, New York, USA; 3 Department of Pathology, University of Virginia, Charlottesville, USA

**Keywords:** dermatopathology, malignancy, cancer, hiv aids, kaposi sarcoma

## Abstract

We present the case of a 68-year-old man with no known risk factors for HIV infection who developed a new, rapidly growing lesion on the left medial foot. The lesion was biopsied and found to be consistent with Kaposi sarcoma (KS). He subsequently tested positive for HIV and developed cellulitis of the left lower extremity. Treatment involved empiric antibiotics, surgical excision of the lesion, radiation therapy, and antiretroviral therapy. The development of KS with no known history of HIV/AIDS is uncommon, with only a few reported cases. We provide a summary of 18 cases in the current literature of cutaneous KS as an initial presenting sign of HIV/AIDS.

## Introduction

Kaposi sarcoma (KS) is a vascular tumor caused by Kaposi sarcoma herpesvirus/human herpesvirus 8 (KSHV/HHV8), which typically presents as multiple violaceous macules, plaques, or nodules involving the face or lower extremities. Less commonly, it may affect the trunk or groin, and in rare cases, it can disseminate systemically [1]. Kaposi sarcoma associated with AIDS (epidemic) most frequently affects HIV-infected men who have sex with men (MSM). The risk of developing AIDS-associated KS is inversely related to CD4+ cell count. An HIV RNA viral load >100,000 copies/mL and CD4 count <200 cells/µL are associated with a higher risk for KS, but up to one-third of cases may present in patients with a non-detectable HIV viral load and a CD4 count greater than 300 cells/µL [2]. Herein, we present the case of a 68-year-old man with a new, rapidly growing lesion involving the skin of the left medial foot diagnosed with KS. He subsequently tested positive for HIV and developed cellulitis of the left lower extremity.

## Case presentation

A 68-year-old man with a distant history of bone spurs and limited access to primary care presented to the outpatient dermatology clinic with a new raised lesion on the left foot. He was first seen in the emergency department approximately two weeks prior, where the lesion was diagnosed as an epidermal inclusion cyst and treated with a corticosteroid injection with minimal improvement. The lesion was persistent, growing, and tender, with occasional bleeding. The patient reported that he had previously experienced bone spurs in the affected region, for which he had undergone corticosteroid injections several years prior. He denied any known personal or family history of skin cancer. He stated that his last HIV test was collected over two years ago, and the results were reportedly negative (test type and reference values unknown). The patient denied sexual activity for the past nine years but endorsed a previous history of unprotected sex with multiple female partners. The patient denied a history of intravenous drug use or blood product transfusions.

**Figure 1:**
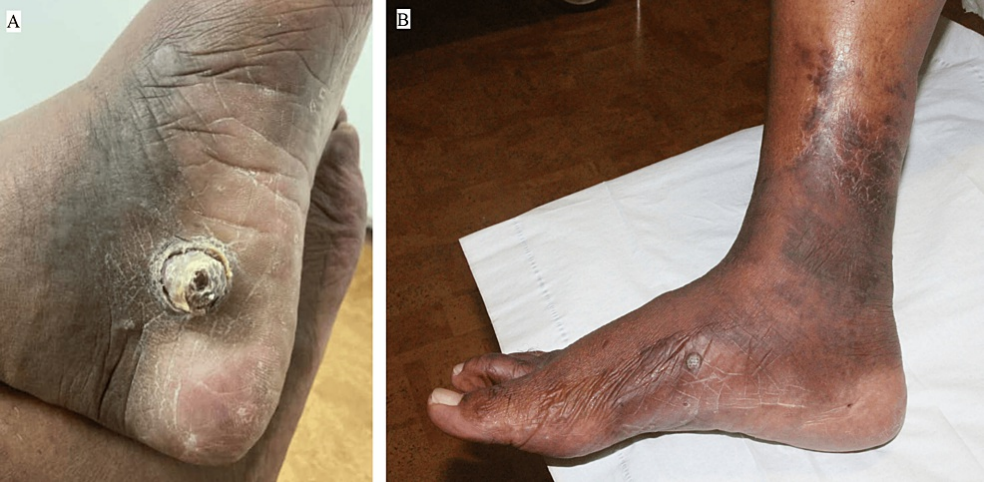
Left medial foot on initial presentation with a 2x2.1 cm firm, circular, exophytic, dry, hyperkeratotic nodule on a pink erythematous base with a collarette of scale (A); violaceous plaques and a hyperpigmented papule on the right medial foot and ankle (B).

On physical examination, a 2x2.1 cm firm, circular, exophytic, dry, hyperkeratotic nodule with a pink erythematous softer base and collarette of scale was noted on the left medial foot (Figure 1A).

On the right medial foot and ankle, there was a hyperpigmented papule with violaceous plaques (Figure 1B). No other cutaneous or oral mucosal lesions were noted. Squamous cell carcinoma, eccrine porocarcinoma, bacillary angiomatosis, drug eruption, pyogenic granuloma, hemangioma, angiodermatitis, coccidioidomycosis, lichen planus, and pseudo-KS were considered in the differential diagnosis.

**Figure 2:**
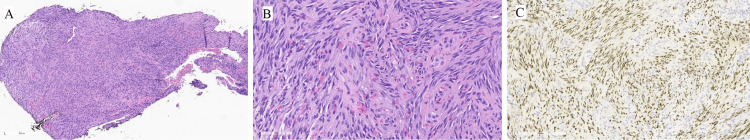
Histopathologic examination revealed dermal proliferation of atypical spindle cells associated with slit-like vascular spaces (A, H&E, 100x). Higher magnification demonstrated nuclear hyperchromasia, scattered mitoses, and extravasated erythrocytes (B, H&E, 200x). The tumor cells were positive for CD34, ERG, and HHV8 (C, 200x). HHV8: human herpesvirus 8

A biopsy of the nodule on the left foot revealed nodular proliferation of atypical spindle cells associated with irregular slit-like vascular spaces and extravasated erythrocytes (Figures 2A, 2B).

The tumor cells were positive for CD34, ERG, and HHV-8, which supported the diagnosis of KS (Figure 2C). Staining for cytokeratin AE1/AE3, S100, and SOX10 was negative. Testing for HIV was positive (CD4+ cell count: 308 cells/µL; viral load: 8,778 copies/mL). Other laboratory investigations, including renal function, liver function, and complete blood count, were within biological limits. Chest radiography findings were unremarkable. The patient was referred to medical oncology and infectious diseases for further management.

**Figure 3:**
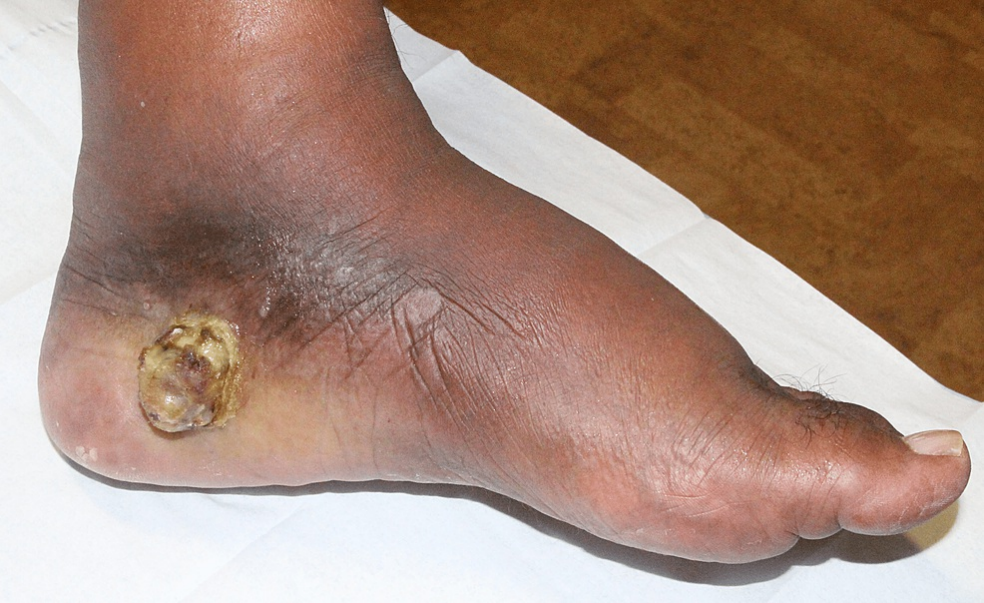
Left medial foot with a purulent, necrotic, exophytic nodule. Darkening of the skin was seen surrounding the nodule, along with moderate diffuse swelling over the dorsal foot and ankle. This image was taken one month after the initial presentation and prior to the surgical excision of the nodule.

Approximately one month later, the patient developed diffuse swelling over the dorsal left foot and ankle. On clinical examination, a large 2x2x2 cm lesion on the medial aspect of the left rear foot was found tender to palpation with foul odor, necrotic tissue, and edema (Figure 3).

He was hospitalized with a primary diagnosis of cellulitis of the left lower extremity and started on empiric antibiotics with piperacillin-tazobactam 4.5 g orally every eight hours for two days and vancomycin 1 g once intravenously. Tissue culture revealed the growth of *Pseudomonas aeruginosa* and *Klebsiella pneumoniae*. During admission, the infected nodule was surgically excised, and histopathology re-demonstrated a highly cellular, monomorphic, atypical, spindled neoplasm with red cell extravasation consistent with KS.

Following excision and completion of antibiotics, he was discharged home with referrals to infectious diseases, medical oncology, and radiation oncology. Treatment with bictegravir, emtricitabine, and tenofovir alafenamide was initiated at a dose of 50-200-25 mg orally once daily to be taken indefinitely as highly active antiretroviral therapy (HAART). The patient was scheduled for radiation therapy but did not return for follow-up with radiation oncology or dermatology. He continues to closely follow up with the infectious diseases, medical oncology, and podiatry departments.

## Discussion

The development of KS prior to a known diagnosis of HIV/AIDS is uncommon, with only a few reported cases. An English-language literature review of articles indexed in Medical Literature Analysis and Retrieval System Online (MEDLINE) describing cutaneous KS as the initial presentation of HIV/AIDS was conducted using the following search terms: "Kaposi sarcoma," "HIV," "presenting," and "initial." In total, 272 abstracts were evaluated for inclusion, and 42 abstracts were identified as potentially eligible for review. Cases of KS initially presenting within other organs or as mucocutaneous lesions were excluded (n = 24). A summary of the 18 cases is presented in Table 1.

**Table 1:** Summary of cases of cutaneous Kaposi sarcoma as the initial presentation of HIV/AIDS. NR: not reported; MSM: men who have sex with men; IVDU: intravenous drug use; NMDA: N-methyl-D-aspartate; HAART: highly active antiretroviral therapy

Source	Patient Age /Sex	Location of primary KS lesion	Comorbid conditions on initial presentation	Risk factors for AIDS-associated KS	CD4+ count (cells/µL)	Viral load (copies/µL)	Metastases at the time of presentation	Management
Angulo et al. (1991) [3]	26, 28/M	Penis, scrotum, right lower extremity, and glans penis	Bacteremia, meningitis, pneumonia, and miliary tuberculosis	IVDU	NR	NR	NR	NR
Vaishnani et al. (2010) [4]	26/M	Face, trunk, and bilateral upper and lower extremities	Molluscum contagiosum	Unprotected sexual activity	186	NR	NR	NR
Armstrong et al. (2014) [5]	28/M	Face, chest, and bilateral upper and lower extremities	None	MSM	149	NR	Lungs	HAART
Kaddu-Mulindwa et al. (2020) [6]	30/M	Right knee, left dorsal foot, and right forearm	Anti-NMDA receptor encephalitis	MSM	360	420,000	None	Steroid pulse therapy, plasmapheresis, immunoadsorption, anti-CD20 therapy, HAART
Weinstein et al. (2016) [7]	30s/M	Right lower eyelid and trunk	None	NR	76	NR	Lungs	HAART, paclitaxel, external beam radiation
Minhas et al. (2008) [8]	31, 38/M	Medial foot and right plantar foot	None	NR	NR	NR	NR	NR
Rapaka et al. (2015) [9]	33/M	Right ankle and right foot	Herpes zoster infection, herpes labialis	MSM	774	28,000	Right external iliac chain lymph nodes	HAART
Tammam et al. (2022) [10]	35/M	Glans penis	None	NR	8	200,000	Lungs	HAART, doxorubicin
Kharkar et al. (2009) [11]	38/M	Bilateral lower extremities	Tuberculosis	NR	115	NR	NR	NR
Teixeira et al. (2016) [12]	40/F	Left upper and lower eyelids	None	NR	88	128,700	Lungs	HAART, doxorubicin
Mehta et al. (2011) [13]	40/M	Bilateral upper and lower extremities, and the trunk	None	Unprotected sexual activity	173	NR	NR	NR
Liu et al. (2006) [14]	43/M	Trunk and the hard palate	None	MSM	289	177,000	Lungs	HAART, doxorubicin
Martorano et al. (2015) [15]	45/M	Bilateral lower extremities	None	NR	NR	NR	NR	NR
Staps et al. (2007) [16]	55/M	Bilateral upper and lower extremities and the trunk	Recurrent upper respiratory infections	MSM	NR	20,000	NR	HAART
Warpe et al. (2014) [17]	65/M	Bilateral upper and lower extremities and the trunk	None	NR	239	NR	NR	NR
Scheinfeld (2004) [18]	NR/M	Left cheek	None	NR	NR	NR	NR	NR

The mean age at diagnosis in the reviewed cases was 37 years (range: 26-65, SD +/- 10.5 years), with a predilection for males (17:1, M:F). The most common location of KS lesions was the lower extremities, and the most common site of metastasis at the time of presentation was the lungs. Reported treatments were HAART with cytotoxic chemotherapy agents [3-18].

The incidence of superimposed cellulitis in KS lesions has not been well described in the literature. However, in a retrospective cohort study of 39 male patients with KS (mean age: 33.7 years; range: not reported, SD+/- 9.5 years), chronic lower limb lymphedema was identified as a risk factor for the development of cellulitis with severe complications. Unilateral lower extremity involvement was reported in 77% of the patients. Other risk factors for the development of cellulitis in KS patients included groin KS infiltration or local lymph node enlargement (69.2%), onychomycosis and/or tinea pedis (44.7%), and an ulcerated KS lesion at the involved limb (38.4%) [19, 20]. Recurrent episodes of cellulitis and hospitalization may be prevented with antibiotic prophylaxis [19].

## Conclusions

Kaposi sarcoma is a reticuloendothelial tumor associated with KSHV/HHV8 infection. AIDS-associated KS is an aggressive variant that typically occurs in untreated or late-stage HIV/AIDS patients with a low CD4+ count. A review of the current literature on KS as the initial presenting sign of HIV/AIDS indicates that the condition presents, on average, in the fourth decade of life and most commonly in males. In these cases, metastases to the lungs were most often reported, suggesting the need to conduct a timely and thorough workup for visceral KS after the diagnosis of cutaneous KS. This is particularly important when HIV status is unknown at the initial presentation.

Without other major comorbidities, known risk factors, or associated systemic symptoms, dermatologists are most likely to evaluate and diagnose cutaneous KS. A multidisciplinary approach between dermatology, medical oncology, and infectious diseases is essential to treating KS with a combination of local and systemic therapies, including HAART.
